# Detecting the genetic basis of local adaptation in loblolly pine (*Pinus taeda* L.) using whole exome‐wide genotyping and an integrative landscape genomics analysis approach

**DOI:** 10.1002/ece3.5225

**Published:** 2019-05-29

**Authors:** Mengmeng Lu, Carol A. Loopstra, Konstantin V. Krutovsky

**Affiliations:** ^1^ Department of Ecosystem Science and Management Texas A&M University College Station Texas; ^2^ Molecular and Environmental Plant Sciences Program Texas A&M University College Station Texas; ^3^ Department of Forest Genetics and Forest Tree Breeding Georg‐August‐University of Göttingen Göttingen Germany; ^4^ Laboratory of Population Genetics, N. I. Vavilov Institute of General Genetics Russian Academy of Sciences Moscow Russia; ^5^ Laboratory of Forest Genomics, Genome Research and Education Center, Institute of Fundamental Biology and Biotechnology Siberian Federal University Krasnoyarsk Russia; ^6^ Present address: Department of Biological Sciences University of Calgary Calgary Alberta Canada

**Keywords:** adaptive phenotypic traits, climate change, environmental association, *F*_ST_ outlier, redundancy analysis, SNP

## Abstract

In the Southern United States, the widely distributed loblolly pine contributes greatly to lumber and pulp production, as well as providing many important ecosystem services. Climate change may affect the productivity and range of loblolly pine. Nevertheless, we have insufficient knowledge of the adaptive potential and the genetics underlying the adaptability of loblolly pine. To address this, we tested the association of 2.8 million whole exome‐based single nucleotide polymorphisms (SNPs) with climate and geographic variables, including temperature, precipitation, latitude, longitude, and elevation data. Using an integrative landscape genomics approach by combining multiple environmental association and outlier detection analyses, we identified 611 SNPs associated with 56 climate and geographic variables. Longitude, maximum temperature of the warm months and monthly precipitation associated with most SNPs, indicating their importance and complexity in shaping the genetic variation in loblolly pine. Functions of candidate genes related to terpenoid synthesis, pathogen defense, transcription factors, and abiotic stress response. We provided evidence that environment‐associated SNPs also composed the genetic structure of adaptive phenotypic traits including height, diameter, metabolite levels, and gene transcript abundance. Our study promotes understanding of the genetic basis of local adaptation in loblolly pine and provides promising tools for selecting genotypes adapted to local environments in a changing climate.

## 1 INTRODUCTION

**Figure 1:**
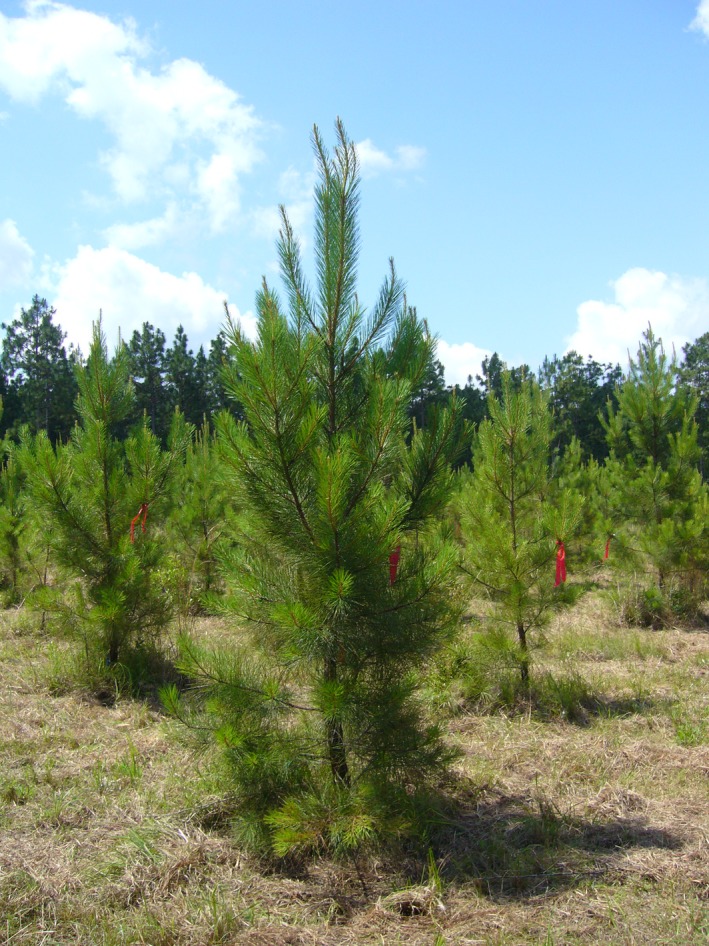
Loblolly pine trees (*Pinus taeda* L.) in the Harrison Experimental Forest at the Southern Institute of Forest Genetics, near Saucier, Mississippi

Loblolly pine (Figure 1) comprises over one half of the standing pine volume in the Southern United States (Baker & Langdon, 1990). The natural habitat of loblolly pine ranges from East Texas to central Florida and north to Southern New Jersey, demonstrating adaptability to various types of soil and growing conditions. Successful forest plantations rely on the selection of appropriate seed sources. The seed transfer guidelines based on climatic modeling of data between the seed source origin and the planting site for southern pines emphasize three key points: (a) low temperature to the north and low rainfall to the west limit the distribution of southern pines; (b) the annual average minimum temperature is the most important climate variable related to growth and survival; (c) for loblolly pine, seeds from east of the Mississippi River should not be used in the west because of the higher danger of losses due to droughts (Schmidtling, 2003).

As the climate changes, traditional seed selection guidelines may need to be adjusted in response to a changing climate scenario. An altered temperature and precipitation pattern threatens forests with droughts, fires, and pathogen outbreaks, eventually leading to damage to the quality and yield of wood produced (Allen et al., 2010). Landscape genomics methods have been applied to explore the genetic basis of local adaptation in loblolly pine. The main objectives of these studies were to identify the environmental factors that have shaped the adaptive genetic variation and the gene variants that drive local adaptation (Rellstab, Gugerli, Eckert, Hancock, & Holderegger, 2015; Sork et al., 2013). Eckert, Heerwaarden, et al. (2010a) found five loci correlated with aridity and identified 24 loci as *F*_ST_ outliers in loblolly pine. Eckert, Bower, et al. (2010) also found several well‐supported loblolly pine SNPs associated with principal components corresponding to geography, temperature, growing degree‐days, precipitation, and aridity. Chhatre, Byram, Neale, Wegrzyn, and Krutovsky (2013) detected SNPs as candidates for diversifying and balancing selection in natural and breeding loblolly pine populations in East Texas. Despite application of multiple methods, the size and complexity of conifer genomes limit the progress to further dissect the genetic basis of local adaptation.

In the current study, we aimed to discover more loci and genes with signatures of natural selection and incorporated phenotypic data into environmental adaption analyses to improve insight. We have discovered 2.8 million SNPs using whole exome sequencing from a clonally propagated association mapping loblolly pine population (Lu et al., 2016, 2017a, 2017b). This population represented diverged ecophysiological regions across 12 states in the Southern United States, extending from Texas to Virginia. Loblolly pine populations have shown adaptation to environment based on the geographic distributions of traits. For example, loblolly pines from west of the Mississippi River are slower growing, but more resistant to fusiform rust, drought, and crowding than trees from east of the Mississippi River (Schmidtling & Froelich, 1993; Wells, 1985). We examined associations of 2.8 million whole exome‐based SNPs with climate and geographic variables in 328 loblolly pine trees using a landscape genomics approach integrating multiple analysis methods. We detected SNPs associated with both adaptive phenotypic traits and climate/geographic variables and identified candidate genes that contribute to local adaptation in loblolly pine. The results can help determine how selection affects the genetic architecture of adaptive traits. The identified loci and genes can contribute to rapid selection of genotypes with adaptive potential to climate change.

## 2 MATERIALS AND METHODS

### 2.1 Plant materials and genotypic data

The loblolly pine trees used in this study were originally collected for the “Allele Discovery of Economic Pine Traits 2” project (ADEPT2) (Cumbie et al., 2011). Maternal trees selected to represent a wide range of the Southeastern United States were open‐pollinated in the local seed orchards. The seeds were grown and used for ADEPT2 project. In the spring of 2010, rooted cuttings of 384 trees from ADEPT2 project were planted in the Harrison Experimental Forest at the Southern Institute of Forest Genetics, near Saucier, Mississippi. We used exome capture method to genotype trees in this population (Lu et al., 2016). Raw SNPs were filtered to retain a total of 2,822,609 SNPs that are biallelic sites without missing data (Lu et al., 2017a, 2017b). For the current study, we analyzed 328 trees with a clearly known county of origin.

### 2.2 Climate and geographic data

Climate and geographic data for counties of origin of each tree in the population were the same as in Eckert, Heerwaarden, et al. (2010a). The data were originally gathered from the WORLDCLIM 2.5‐min geographical information system (GIS) layer using Diva‐GIS v.5.4 (Hijmans, Cameron, Parra, Jones, & Jarvis, 2005). The dataset contained a total of 58 variables, including latitude, longitude, elevation, average minimum and maximum temperature for each month, average precipitation for each month, and 19 bioclimatic variables (Table S1). The bioclimatic variables are summary statistics of precipitation and temperature. For example, BIO1 represents annual mean temperature, and BIO12 represents annual precipitation. The JMP Pro 12 statistical software (SAS Institute) was used to display the variation of climate variables across the counties of origin for the studied trees.

### 2.3 Environmental associations and outlier analyses

**Figure 2:**
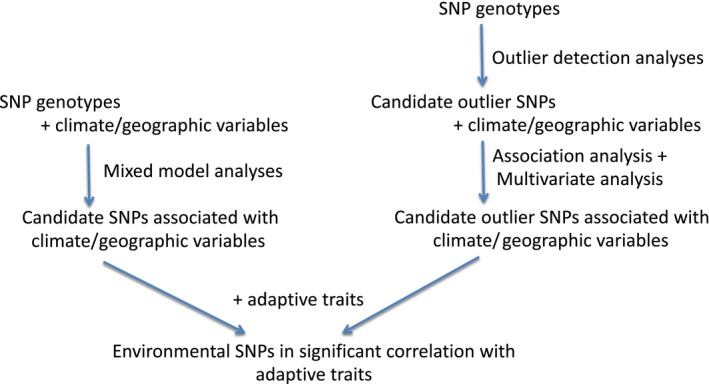
Summary of different approaches used in this study

Multiple approaches were employed to discover the loci associated with climate and geographic variables. The process is schematically summarized in Figure 2. We associated 2.8 million SNPs with climate/geographic variables using TASSEL 5.0 (Bradbury et al., 2007). We applied general linear model (GLM) method (*S* model), mixed linear model (MLM) method incorporating a kinship matrix (*K* model), the GLM incorporating the covariate to adjust for population structure (*Q* model) and the MLM incorporating both the kinship matrix and population structure covariate (*QK* model) to conduct association analyses. Simple sequence repeat (SSR) markers generated from the previous study (Eckert, Heerwaarden, et al., 2010a) were used to adjust for population structure. However, the SSR markers were only available for 249 out of the total 328 trees. We grouped the 249 trees with SSR markers as *pop* population. Since the genetic structure of this population is mainly caused by the Mississippi River, we grouped the 300 trees in the east of the Mississippi River as *east* population. We did not analyze the trees in the west of the Mississippi River because there are too few trees. The total 328 trees were called *total* population. We applied *S* and *K* models to the *total* and *east* populations. We applied *S*, *K*, *Q,* and *QK* models to the *pop* populations. The kinship matrix was estimated using the SNP markers of each population and the TASSEL 5.0 (Bradbury et al., 2007). Quantile–quantile plots were generated for observed against expected −log_10_ (*P*) to examine the model fitness. Significance of associations between loci and traits were determined by the *p‐*values. A corrected Bonferroni threshold 0.05/94,478 = 5.29E‐7, where 94,478 was the number of haplotype blocks in the studied population (Lu et al., 2017a), was applied to screen for significant loci. The intersection of the SNPs acquired by different models was used in the downstream analyses.

Two outlier detection methods were employed to detect loci under selection and potentially involved in local adaption. One method is the spatial ancestry analysis (SPA), which identifies SNPs with significant gradients in allele frequency (Yang, Novembre, Eskin, & Halperin, 2012). The geographical location (longitude and latitude) information for each tree was supplied as the “—location‐input.” SNPs with SPA scores above the 99.9% percentile were considered as outliers. Another outlier detection method was implemented by the OutFLANK software (Whitlock & Lotterhos, 2015). It infers the *F*_ST_ distribution for a large set of loci and identifies the loci that may contribute to a significant local differentiation and potential adaptation. A *Q*‐value of 0.05 was applied to detect outliers. Following the program recommendation, 1,323,910 SNPs with a minor allele frequency (MAF) ≥0.05 were used for the SPA and OutFLANK analyses.

We used multivariate analysis to identify the significance of climate in structuring genetic diversity among the outlier SNPs. The multivariate relationships were examined using the redundancy analysis (RDA) implemented by the R package *vegan* (Oksanen et al., 2017; R Core Team, 2018). We examined the SNP outliers’ variation explained by pure climate, or pure geography variables, or both climate and geography variables using RDA (Sork et al., 2016). We first ran a full RDA model by using all climate and geographical variables as explanatory variables and SNP outliers as dependent variables. The total explainable variance is the inertia (variance) for the constrained matrix of the full model. Then we ran a partial RDA (pRDA) model by using climate as explanatory variables, which were conditioned on geography, and SNP outliers as dependent variables. The pure climate variance is the inertia for the constrained matrix conditioned on geography. We also ran a pRDA model by using geography as explanatory variables, which were conditioned on climate, and SNP outliers as dependent variables. The pure geography variance is the inertia for the constrained matrix conditioned on climate. The joint climate/ geography variance was calculated by subtracting the pure effects from the total explainable variance. Statistical significance of the model estimates was assessed using a permutation‐based analysis of variance (ANOVA).

Association of the outlier SNPs with climate and geographic variables was analyzed using the Samβada software (Stucki et al., 2017). This software is based on the logistic regression model and assesses whether the allelic variation correlates with specific environmental variables. Spatial association due to population structure is accounted for by measuring indices of spatial autocorrelation of geographical coordinates of each tree. In this study, the parameters for Samβada analysis were set up as: Spatial autocorrelation was measured along longitude and latitude using spherical coordinate and 20 nearest neighbors; both global and local autocorrelation of loci were included, and the significance was assessed with 1,000 permutations. The detection of selection signatures was based on univariate models, and the threshold for screening significant models was set to 1%.

We searched for SNPs associated with both adaptive phenotypic traits and climate/geographic variables to better understand how selection pressures shape the genetic structures underlying local adaptation. Using the SNP set of the current study, we previously found SNP associations with such adaptive phenotypic traits as specific leaf area, branch angle, height, diameter, crown width, carbon isotope discrimination, and nitrogen content (Lu et al., 2017a). Briefly, we sampled and measured the traits of the studied loblolly pine trees at the Harrison Experimental Forest in the spring of 2014. We measured height, branch angle, stem diameter, and crown width on the site. We collected needle samples and measured specific leaf area, carbon isotope discrimination, and nitrogen content in the laboratory. We also found SNP associations with metabolite levels and expression of wood development‐ and stress resistance‐related genes (Lu, Seeve, Loopstra, & Krutovsky, 2018). Relative transcript abundance of 111 genes involved in xylem development (Palle et al., 2011) and 88 genes involved in disease or drought response (Seeve, 2010) were measured using reverse transcription–quantitative polymerase chain reaction (RT‐qPCR). A total of 82 metabolites with known names were measured in woody tissues (Eckert et al., 2012). By associating 2.8 million exome‐based SNPs and adaptive phenotypic traits, we identified 5 SNPs associated with specific leaf area, 2 with branch angle, 3 with crown width, 4 with stem diameter, 9 with total height, 4 with carbon isotope discrimination, 2 with nitrogen concentration, 1841 with 191 gene expression mRNA phenotypes, and 524 with 53 metabolite level phenotypes. In the current study, we focused on the loci associated with both adaptive phenotypic traits and climate/geographic variables. The JMP Pro 12 statistical software (SAS Institute) was employed to display the variation of climate/geographic variables, genotypes, and phenotypic traits.

We obtained the annotation for genes that contain identified SNPs from loblolly pine gene annotation files available on https://treegenesdb.org/FTP/Genomes/Pita/v1.01/annotation/ (Wegrzyn et al., 2014). SNPs within 5,000 bp downstream or upstream of a gene were considered to be within a putative regulatory sequence. If a SNP is located in a region without annotation, we performed a blastx search by querying the flanking sequence 700 bp upstream and downstream of the SNP against the entire National Center for Biotechnology Information (NCBI) nonredundant (nr) protein database (http://blast.ncbi.nlm.nih.gov/Blast.cgi). The VCFtools software (Danecek et al., 2011) was used to calculate the minor allele frequency (MAF).

## 3 RESULTS

### 3.1 Climate variation in the loblolly pine natural range

**Figure 3:**
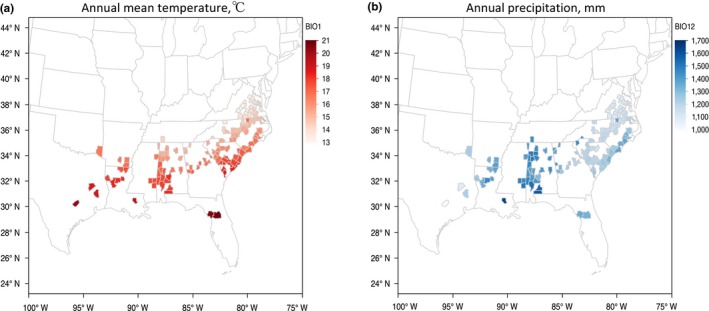
Variation in climate variables BIO1 (a) and BIO12 (b) across counties of origin

Among the counties of origin for the studied trees, the annual mean temperature (BIO1) demonstrated a decreasing trend from South to North (Figure 3a). The annual precipitation (BIO12) was higher in Louisiana, Mississippi and Alabama than in other regions (Figure 3b). Maximum temperature of the warmest month (BIO5) and mean temperature of the driest quarter (BIO9) were higher in the western range (Figure S1). Mean temperature of the wettest quarter (BIO8), precipitation seasonality (BIO15), and precipitation of wettest and warmest quarter (BIO16 & BIO18) were higher in the eastern range (Figure S2). Precipitation of the coldest quarter (BIO19), driest month (BIO14), and driest quarter (BIO17) were higher in Louisiana, Mississippi and Alabama compared with other states. Along South to North, minimum temperature of the coldest month (BIO6) and mean temperatures of the warmest and coldest quarters (BIO10 & BIO11) decreased, while temperature seasonality (BIO4) and annual temperature range (BIO7) increased.

### 3.2 SNPs associated with climate and geographic variables

We identified 503 associations, including 49 climate/geographic variables and 293 SNPs (Table S2). Among them, 297 associations involved temperature variables, 174—precipitation variables, 21—elevation, and 11—latitude. The MAF of the identified SNPs were between 0.01 and 0.5 with a median of 0.02. Among the 293 SNPs, 199 were in 195 annotated genes. Specifically, 3 SNPs (2%) were in 3′ regulatory sequences (3′ RS), 9 (4%) in 5′ RS, 118 (59%) in coding sequences (CDS), 59 (29%) in introns, 5 (3%) in 5′ untranslated regions (5′ UTR), and 5 (3%) in 3′ UTR. The remaining SNPs were in unclassified or intergenic regions. Most identified SNPs were associated with multiple variables. For example, the SNP tscaffold3881_229913 was associated with latitude, 3 precipitation variables, and 25 temperature variables. This SNP resides in the CDS of a gene encoding EARLY FLOWERING 3‐like protein, which is a circadian clock protein playing key roles in adaptation of plants to diurnal environmental conditions.

### 3.3 Outlier SNPs

We found that 1,324 SNPs showed large gradients in allele frequency based on the SPA analysis (Table S3). Among them, 1,099 SNPs resided in 381 annotated genes. Specifically, 43 SNPs (4%) resided in 3′ RS, 68 (6%) in 5′ RS, 548 (50%) in CDS, 380 (35%) in introns, 14 (1%) in 5′ UTR, and 46 (4%) in 3′ UTR. The other SNPs resided in unclassified or intergenic regions. The annotated genes PITA_000021128 and PITA_000021125 contained the most outlier SNPs, 38 and 27, respectively. These two genes encode the ent‐copalyl diphosphate synthase and the abietadienol/abietadienal oxidase‐like protein, respectively. Both genes participate in terpenoid synthesis and contribute to conifer defense against herbivores and pathogens.

We also identified 242 SNP outliers using the OutFLANK software (Table S4). Among them, 189 SNPs resided in 128 annotated genes. Specifically, 8 SNPs (4%) resided in 3′ RS, 11 (6%) in 5′ RS, 120 (64%) in CDS, 44 (23%) in introns, 2 (1%) in 5′ UTR, and 4 (2%) in 3′ UTR. The remaining SNPs resided in unclassified or intergenic regions. The annotated genes PITA_000091177, PITA_000064023, and PITA_000040532 contained the most outlier SNPs. These three genes encode a LRR receptor‐like serine/threonine‐protein kinase, a bHLH transcription factor, and a protein of unknown function.

We found 33 loci identified by both SPA and OutFLANK software (Table S5). The MAFs of these 33 loci ranged between 0.06 and 0.47 with a median of 0.21. These 33 loci resided in 12 annotated genes encoding proteins that include the leucine‐rich repeat receptor‐like serine/threonine‐protein kinase, the bHLH transcription factor, oxidoreductase, and an EARLY FLOWERING 3‐like protein.

### 3.4 Multivariate analyses of the identified SNP outliers

The multivariate analyses using redundancy analysis (RDA) confirmed that the outlier SNPs are significantly correlated (ANOVA *p* < 0.0) with climate when conditioned on geography. Pure climate explained 51% of the SNP outliers' variance. The outlier SNPs are insignificantly (ANOVA *p* = 0.6) correlated with geography when conditioned on climate. Pure geography explained 2% of the SNP outliers' variance. However, the remaining proportion of SNP outliers' variance (47%) was rather large due to the joint effect of climate and geography, demonstrating their interactive influence on the SNP variation. For the partial RDA that analyzed relationship between outliers SNPs and climate variables conditioned on geography, the first and second constrained axes (RDA1 and RDA2) explained the most SNP outliers' variance (15% and 8%, respectively, Table S6). The variables explained the most variation on RDA1 were average precipitation in January, February, March, April, and December, precipitation of coldest quarter (BIO19), precipitation of the driest quarter (BIO17), mean temperature of wettest quarter (BIO8), mean diurnal range (BIO2), and precipitation of driest month (BIO14) (Figure S3 and Table S7).

### 3.5 Outlier SNPs associated with climate and geographic variables

We identified 1,790 associations between 323 SNP outliers and 47 climate/geographic variables using the Samβada software (Table S8). Among them, 963 associations were related to temperature, 476 to precipitation, 41 to latitude, and 310 to longitude. The outlier SNPs associated with environment had MAFs between 0.05 and 0.49 with a median of 0.21, residing in 250 annotated genes.

**Figure 4:**
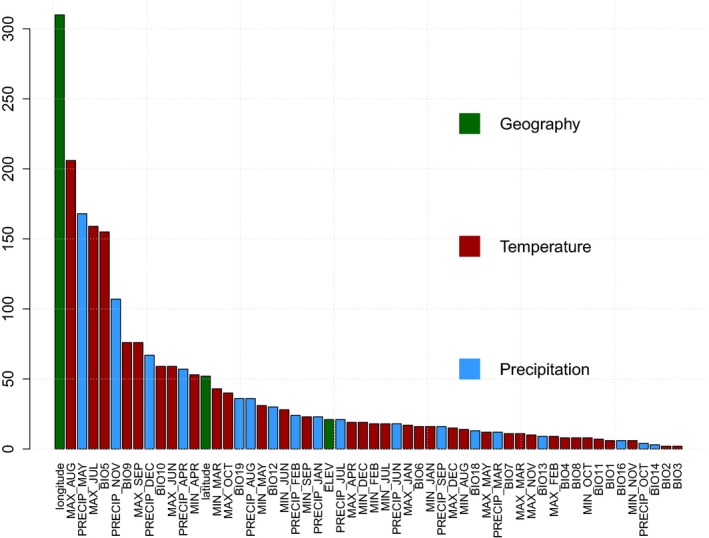
Numbers of environmental SNPs (envSNPs) associated with geographic and climate variables. Red bars represent temperature variables. Blue bars represent precipitation variables. Green bars represent geographic variables. MAX and MIN represent maximum and minimum temperature of each month. PRECIP represents average precipitation of each month. BIOs represent 19 bioclimatic variables. Details of BIOs are listed in Table S1

Taken together, we identified 611 unique SNPs associated with 56 climate and geographic variables (“environmental SNPs”—envSNPs) using either the TASSEL or Samβada software (Table S9). Only two variables, precipitation seasonality (BIO15) and precipitation of the driest quarter (BIO17) were not found to be associated with any SNP. Of the other variables, longitude was associated with the most SNPs (310), followed by maximum temperature of August (206), precipitation of May (168), maximum temperature of July (159), maximum temperature of the warmest month (BIO5) (155), precipitation of November (107), maximum temperature of September (76), mean temperature of the driest quarter (BIO9) (76), precipitation of December (67), maximum temperature of June (59), and mean temperature of the warmest quarter (BIO10) (59) (Figure 4). Among the 611 envSNPs, 248 of them reside in CDS, including 159 nonsynonymous envSNPs, and 89 synonymous envSNPs (Table S9). 14 nonsynonymous envSNPs cause stop codon, residing in genes encoding mitogen‐activated protein kinase, terpene synthase‐related protein, and late embryogenesis abundant protein. Nonetheless, nonsynonymous envSNPs were not significantly over‐represented in the candidate genes according to Fisher's exact test (*p* = 0.2).

We found the genes containing the 611 envSNPs mostly related to resistance to abiotic and biotic stresses; thus, we categorized these genes into four main functional groups: (a) terpenoid synthesis, (b) pathogen and disease defense, (c) transcription factors, and (d) abiotic stress response (Table 1 and Table S10). Among the 611 envSNPs, five SNPs (scaffold10517.2_56785, scaffold674735_1427, scaffold721455_39357, tscaffold3881_229913, and tscaffold551_336950) were detected by both TASSEL and Samβada software. They resided in the following four annotated genes: PITA_000048497, PITA_000060878, PITA_000004436, and PITAhm_001489, which encode an abietadienol/abietadienal oxidase‐like protein, a myrcene synthase or terpene synthase metal‐binding domain protein, an EARLY FLOWERING 3‐like protein, and a DEAD/DEAH box helicase domain protein.

**Table 1:** Four main functional groups of genes with SNPs associated with climate and geographic variables

Biological functional groups	Gene function
Terpenoid synthesis	Myrcene synthase, malate synthase, cytochrome P450, shikimate O‐hydroxycinnamoyltransferase, (−)‐alpha‐pinene synthase, (−)‐alpha‐terpineol synthase
Pathogen and disease defense	LRR receptor‐like serine/threonine‐protein kinase, cucumisin‐like serine protease, benzyl alcohol O‐benzoyltransferase, mitogen‐activated protein kinase homolog MMK2
Transcription factor	bHLH, MADS, MYB, GRAS
Abiotic stress response	Asparagine synthetase, 2‐oxoisovalerate dehydrogenase, late embryogenesis abundant protein, WAT1‐related protein, bark storage protein A‐like

Note

A full list of genes containing SNPs associated with climate and geographic variables is presented in Table S10.

### 3.6 SNPs associated with both climate/geographic variables and adaptive phenotypic traits

We identified five envSNPs associated with both height and diameter, 10 with height only, 114 with 27 metabolite levels, and 242 with expression levels of 47 genes (Table 2 and Tables S11 and S12). For example, 54 envSNPs associated with arachidic acid levels, and more than 60 envSNPs associated with the expression levels of *ANR* and *NCED* genes.

**Table 2:** SNPs associated with both adaptive phenotypic traits (height and diameter) and climate/geographic variables

SNP	Trait	Climate or geographic variable	MAF	Gene	Location	Annotation
C31450188_4342	H, D	MAX_JAN‐FEB, MAX_NOV‐DEC, MIN_MAR‐JUN, MIN_AUG‐SEP	0.31	Unknown	NA	Protein detoxification^a^
C31663680_2810	H, D	MAX_JAN, MAX_DEC	0.01	Unknown	NA	Aspartyl protease, family protein^a^
C32551668_111344	H	Longitude, MAX_AUG, PRECIP_MAY	0.08	PITA_000045229	CDS	MYC‐type basic helix‐loop‐helix(bHLH) domain
scaffold141845.2_19879	H	BIO19, PRECIP_JAN	0.13	PITA_000039839	CDS	Carotenoid cleavage dioxygenase
scaffold16028.2_21200	H, D	MAX_JAN, MAX_DEC	0.01	PITA_000079070	CDS	Transcription factor GRAS
scaffold291933_21926	H, D	MIN_MAR‐SEP	0.24	PITA_000071412	intron	No apical meristem family protein
scaffold478810_88721	H	PRECIP_APR	0.08	PITA_000046000	3'UTR	LOB domain‐containing protein
scaffold785666_22438	H	MIN_MAR‐MAY, MIN_SEP	0.03	PITA_000071386	CDS	Pentatricopeptide repeat‐containing protein
scaffold852567.1_4187	H	MIN_ MAR‐SEP	0.43	Unknown	NA	Unknown^a^
scaffold898461_41654	H	BIO5, longitude, MAX_JUL‐AUG	0.16	PITA_000071959	CDS	Oxidoreductase, 2OG‐Fe(II) oxygenase family protein
tscaffold3336_94487	H	PRECIP_JUN	0.07	PITAhm_000419	intron	Cationic peroxidase 1‐like
tscaffold3355_135785	H	MIN_SEP, PRECIP_SEP	0.04	Unknown	NA	Clavaminate synthase‐like^a^
tscaffold3599_564571	H	PRECIP_JUN	0.01	PITAhm_000122	intron	FACT complex subunit SSRP1
tscaffold3881_229913	H	Latitude, BIO4, BIO6, BIO7, BIO11, BIO18, MAX_JAN‐MAY, MAX_SEP‐DEC; MIN_JAN‐DEC	0.47	PITA_000004436	CDS	Protein early flowering 3‐like
tscaffold7920_235666	H, D	MIN_MAR	0.01	PITA_000025194	CDS	Transmembrane 9 superfamily member

Note

SNPs were named using scaffold name followed by the SNP position in the scaffold.

Abbreviation(s): 3′ UTR, 3′ untranslated regions; BIOs, 19 bioclimatic variables detailed in Table S1; CDS, coding sequences; D, diameter measured at 18 inches above the ground; H, height; MAF, minor allele frequency; MAX and MIN, maximum and minimum temperature of each month; NA, not annotated; PRECIP, average precipitation of each month.

a For SNP that was not located in the annotated region, the flanking sequence 700 bp upstream and downstream of the SNP was used to query against the NCBI Genbank nonredundant protein database using blastx.

**Figure 5:**
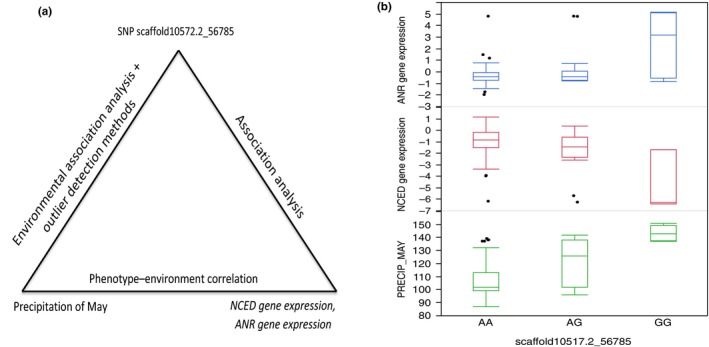
Example of combining SNPs, climate/geographic variables, and adaptive phenotypic traits to analyze the genomic basis of local adaptation in loblolly pine. (a) The A/G SNP scaffold10517.2_56785 resides in a gene encoding the abietadienol/abietadienal oxidase‐like protein. This SNP was identified to be associated with precipitation in May as well as expression levels of *NCED* and *ANR* genes. (b) Precipitation values in May and expression levels of *NCED* and *ANR* genes for the SNP scaffold10517.2_56785 genotypes AA, AG, and GG

We combined genomic, phenotypic, and climate/geographic data to analyze adaptive genetic variation. For example, we found the envSNP scaffold10517.2_56785 (identified by both association and outlier detection methods) correlated with expression levels of the *ANR* and *NCED* genes. The expression levels of these two genes also correlated with precipitation of May (Figure 5a). The *ANR* gene encodes an anthocyanidin reductase, which is important for the biosynthesis of condensed tannins (Xie, Sharma, Paiva, Ferreira, & Dixon, 2003). The *NCED* gene encodes a 9‐*cis* epoxycarotenoid dioxygenase, which prepares precursors for synthesis of abscisic acid (ABA) (Tan et al., 2003). ABA is a key regulator of seed development, root growth, stomatal aperture, and plant responses to water stress. The envSNP scaffold10517.2_56785 resided in a gene encoding an abietadienol/abietadienal oxidase‐like protein, which is a multifunctional and multisubstrate cytochrome P450 monooxygenase that contributes to conifer defense by generating an enormous structural diversity of plant terpenoid secondary metabolites (Ro, Arimura, Lau, Piers, & Bohlmann, 2005). Individuals with the AA genotype tended to have low expression of the *ANR* gene and high expression of the *NCED* gene (Figure 5b). They were common in counties with low precipitation in May (Figure S4). On the contrary, individuals with the GG genotype had high expression of the *ANR* gene and low expression of the *NCED* gene. They were common in counties with high precipitation in May. Individuals with the AG genotype were common in counties with medium precipitation in May, and the expression of the *ANR* and *NCED* genes did not differ much from the individuals with the AA genotypes. Precipitation in May positively correlated with the *ANR* gene expression level (*r* = 0.4, *p* < 0.0) and negatively correlated with the *NCED* gene expression level (*r* = −0.2, *p* = 0.0).

## 4 DISCUSSION

We identified 611 envSNPs associated with 56 climate and geographic variables. Longitude, maximum temperature of the warm months and monthly precipitation associated with most envSNPs. The identified envSNPs resided in genes related to terpenoid synthesis, pathogen and disease defense, transcription factors and abiotic stress response. We also found that some envSNPs composed the genetic structure of adaptive phenotypic traits including height, diameter, metabolite levels, and expression of genes.

### 4.1 Comparison of multiple analysis methods

Combining environmental association analyses with outlier detection methods is a desirable way to reduce the rate of false positives and assess the relevance of findings in landscape genomic research (Le Corre & Kremer, 2012; Rellstab et al., 2015), but each method has its strengths and weaknesses. TASSEL exploits the genomic diversity at a very high resolution; hence, it is sensitive for detecting associations even for SNPs with low minor allele frequencies (MAFs). In this study, among the 293 envSNPs that demonstrated significant associations with climate and geographic variables detected by TASSEL, 72% had a MAF <0.05. Associations could be due to linkage disequilibrium with the functional loci and hence not directly involved in environmental adaptation. The SPA and OutFLANK software detect SNPs under strong selection. To apply these two methods, loci with low MAFs (<0.05) were removed due to a probable high sampling variance, which may negatively affect the power of models. This is especially critical for OutFLANK, because the distribution of *F*_ST_ for loci with low MAFs is very different from that for loci with more equal allele frequencies (Whitlock & Lotterhos, 2015). The MAFs of SNPs detected by SPA ranged from 0.06 to 0.5 with a median of 0.36. The MAFs of SNPs detected by OutFLANK ranged from 0.05 to 0.47 with a median of 0.07. Since most adaptation related traits are polygenic with small allele frequency changes at many loci (Le Corre & Kremer, 2012; Mackay, Stone, & Ayroles, 2009), SPA and OutFLANK would miss those loci under weak selection. Additionally, SPA and OutFLANK cannot identify the specific factors that drive selection. To further determine the selective factors, the Samβada software was applied to associate climate and geographic variables with SNP outliers while taking into account spatial autocorrelation using geographical coordinates of each tree. The Bonferroni correction implemented in the current Samβada software may be overly conservative and may result in overlooking potentially adaptive loci (Stucki et al., 2017). We applied the multivariate approach RDA to examine the relationship between climate/geographic variables and genetic variation of the outlier SNPs. We identified precipitation factors as the important drivers for local adaption. However, the joint effect of climate and geography due to collinearity comprised 47% of the SNP outliers' variance, and the strong pattern of collinearity may skew the results (Rellstab et al., 2015).

The overlap rate among the SNPs detected by different software was relatively low. Among the 293 envSNPs identified by TASSEL and 323 envSNPs identified by outlier methods and Samβada, only 5 envSNPs were the same. The minimal overlap between polygenic alleles detected by environmental association and SNPs outliers detected by the outlier methods is consistent with previous studies on loblolly pines and other species (Anderson, Kono, Stupar, Kantar, & Morrell, 2016; Eckert et al., 2013). The minimal overlap indicated two different modes of local adaptation: selective sweeps and polygenetic adaptation (Pritchard & Di Rienzo, 2010). These two modes may influence different sets of alleles. Selective sweeps drive adaptive alleles to fix in population, while polygenetic adaptation attain adaptation through modest changes in allele frequencies at large numbers of small‐effect alleles (Pritchard, Pickrell, & Coop, 2010). Selective sweeps can occur and impact polygenic adaptation (Stetter, Thornton, & Ross‐Ibarra, 2018), but polygenetic traits rarely reach fixation (Pavlidis, Metzler, & Stephan, 2012), thus most polygenic adaptation alleles with small allele frequency shifts cannot be distinguished from neutral pattern of variation. We need to apply different models to detect the polygenic adaptation and selective sweeps (Pritchard & Di Rienzo, 2010). Another explanation for the minimal overlap is either method used in the current study, association or outlier, or even neither of them, has enough power to detect most adaptive alleles; thus, output of the two methods does not overlap. Large numbers of small‐effect alleles mainly compose the genetic structure of adaptive phenotypes (Mackay et al., 2009; Rockman, 2012). However, small‐effect alleles are prone to homogenizing effects from migration or gene swamping (Lenormand, 2002; Yeaman, 2015), leaving many alleles that contribute to local adaption do not have large differences in allele frequency compared with putatively neutral markers; thus, conventional models are less powerful in detecting small‐effect alleles. Large sample size and improved design may increase the power to discover the polygenic alleles with small effects (Berg & Coop, 2014).

There is no single widely accepted statistical approach (Rellstab et al., 2015). Integrating multiple methods and compiling all possible results can provide more reliable information for downstream analyses. Follow‐ups are needed to validate the detected adaptive loci and genes using independent populations, knockout mutants, common garden, and reciprocal transplant experiments (Rellstab et al., 2015).

### 4.2 Evidence of selection by environment

The identified SNP‐environment associations helped us recognize the climate and geography variables that have shaped the genetic variation. We found that longitude, maximum temperature of the warm months and monthly precipitation were variables associated with the most envSNPs (Figure 4). They may act as selective factors driving loblolly pine local adaptation. Although the seed transfer guidelines advised the yearly average minimum temperature as the most important climate variable for southern pines (Schmidtling, 2003), the current study highlights the importance and complexity of maximum temperature of the warm months and monthly precipitation in shaping the genetic variation underlying loblolly pine adaptability. A significant increase in the number of consecutive days exceeding 35°C (a metric used as a measure of heat waves) and a decline in the net water supply availability are expected over the next decades, particularly in the western part of the loblolly pine range (Kunkel et al., 2013; Sun, 2013). In a rapid climate change scenario, if adaptation of loblolly pine cannot match the increased heat and drought conditions, the productivity and thus the economic and ecological profits will be greatly damaged. Selecting and planting genotypes adapted to the changing climate may reduce losses in loblolly pine plantations. A cautionary note of the current study is that the studied trees were grown in local seed orchards from open‐pollinated seeds. Although the seed orchards were close to the origins of seeds, the uncertainty of paternal origin may leave the studied trees have different adaptive phenotypes from the naturally grown trees.

According to a global assessment, forest trees may be exhibiting increased mortality due to drought, heat, insect outbreaks, and wildfires under climate change (Allen et al., 2010). The identified candidate genes directly or indirectly related to abiotic or biotic stress response, including four functional groups: (a) terpenoid synthesis, (b) pathogen and disease defense, (c) transcription factors, and (d) abiotic stress response (Table 1 and Table S10). For example, genes encoding the myrcene synthase and cytochrome P450 are in the terpenoid biosynthesis pathway. Terpenes offer chemical defense against herbivores and pathogens in conifers. The gene encoding an LRR receptor‐like serine/threonine‐protein kinase is related to pathogen and disease resistance. The transcription factors bHLH and MADS‐box regulate downstream defensive and developmental reactions. Other genes are related to responses to abiotic stresses, including stresses from UV, salt, drought, nitrogen, cold, heat, oxidation, and wounding. These stress response genes contribute to the genetic structure of loblolly pine adaptability, conferring mitigation, and adaptation potential in diverse environments. Five genes related to loblolly pine adaptability and detected in the current study were also reported earlier (Eckert, Heerwaarden, et al., 2010a). These consistently detected genes encode the MATE efflux family protein, a methyltransferase, a translation initiation factor, a ubiquitin, and an auxin responsive protein. They are associated with multiple climate and geographic variables including longitude, monthly precipitation, and average maximum monthly temperature. For example, the gene encoding the MATE efflux family protein was previously identified to correlate with aridity (Eckert, Heerwaarden, et al., 2010a). In the current study, this gene was found to be associated with average maximum temperature in February and March, precipitation in January, February, April, June, November, and December, mean temperature of the driest quarter (BIO9), annual precipitation (BIO12), and precipitation of the coldest quarter (BIO19). The MATE efflux family proteins play important roles in a wide range of biological processes, such as transporting secondary metabolites, regulating disease resistance, and detoxifying toxic compounds (Liu, Li, Wang, Gai, & Li, 2016). These consistently detected genes are strong candidates underlying loblolly pine adaptability.

Combining environmental association analyses with dissection of phenotypic traits can greatly improve our understanding of the genetic basis of local adaptation. Talbot et al. (2017) reported that loci with local adaptation signatures in loblolly pine were also linked to gene expression traits for lignin development and whole‐plant traits. In our study, more associations between loci with local adaption signatures and adaptive phenotypic traits were detected due to the application of 2.8 million SNPs. The loci with local adaption signatures correlated with height, diameter, metabolite levels, and expression of genes. These results indicate that genes underlying adaptive phenotypic traits are likely involved in adaptability to the environment. These candidate genes need to be further tested in validation populations located in different environments.

## 5 CONCLUSION

We identified 611 SNPs associated with 56 climate and geographic variables using an integrative landscape genomics approach by combining association analyses with outlier detection analyses. Longitude, maximum temperature of the warm months, and monthly precipitation associated with most SNPs, indicating their importance and complexity in shaping the genetic variation underlying loblolly pine adaptability. The identified SNPs resided in genes related to terpenoid synthesis, pathogen and disease defense, transcription factors and abiotic stress response. We provided evidence that environment‐associated SNPs (envSNPs) also composed the genetic structure of adaptive phenotypic traits including height, diameter, metabolite levels, and expression of genes. The climate trend in the loblolly pine range, increasing heat and drought, pose challenges for breeding loblolly pine adapted to the planting environment. Our study provides envSNPs and candidate genes to facilitate elucidation of the genetic architecture of environmental adaptation in loblolly pine. The knowledge can be applied in breeding loblolly pine trees adapted to future local environment.

## CONFLICT OF INTEREST

The authors declare no competing interest.

## AUTHOR CONTRIBUTIONS

C.A.L and K.V.K. conceived idea, designed the study, obtained the funding, coordinated the laboratory and field work, and assisted with editing the manuscript. M.L. performed the sample collection, data generation and analyses, and wrote the draft manuscript. All authors read and approved the final manuscript.

## Data Availability

The 2.8 million whole exome‐based SNPs used in this study are available from the Dryad Digital Repository (https://doi.org/10.5061/dryad.269126c).
