# Supplementary material for: Detecting the genetic basis of local adaptation in loblolly pine (Pinus taeda L.) using whole exome‐wide genotyping and an integrative landscape genomics analysis approach
Source: Ecol Evol. 2019 May 29;9(12):6798–809. doi: 10.1002/ece3.5225 (PMC6662259; doi:10.1002/ece3.5225)
Supplement: Supplementary file 1 [file ECE3-9-6798-s001.docx]

**Supporting Information 2 for:**

**Detecting the genetic basis of local adaptation in loblolly pine (*Pinus taeda* L.) using whole exome-wide genotyping and an integrative landscape genomics analysis approach**

**Mengmeng Lu │ Carol A. Loopstra │ Konstantin V. Krutovsky**

**FIGURE S1** Variation of temperature-related bioclimatic variables across counties of origin of the studied loblolly pine trees

**FIGURE S2** Variation of precipitation-related bioclimatic variables across counties of origin of the studied loblolly pine trees

**FIGURE S3** Biplot scores for climate variables on the first constrained axis (RDA1)

**FIGURE S4** Analyses of the SNP scaffold10517.2_56785 and its associated climate variable as well as adaptive phenotypic traits for the studied population

**FIGURE S1** Variation of temperature-related bioclimatic variables across counties of origin of the studied loblolly pine trees (BIO2: Mean diurnal range; BIO3: Isothermality; BIO4: Temperature seasonality; BIO5: Maximum temperature of warmest month; BIO6: Minimum temperature of coldest month; BIO7: Temperature annual range; BIO8: Mean temperature of wettest quarter; BIO9: Mean temperature of driest quarter; BIO10: Mean temperature of warmest quarter; BIO11: Mean temperature of coldest quarter)

**FIGURE S2** Variation of precipitation-related bioclimatic variables across counties of origin of the studied loblolly pine trees (BIO13: Precipitation of wettest month; BIO14: Precipitation of driest month; BIO15: Precipitation seasonality; BIO16: Precipitation of wettest quarter; BIO17: Precipitation of driest quarter; BIO18: Precipitation of warmest quarter; BIO19: Precipitation of coldest quarter)

**FIGURE S3** Biplot scores for climate variables on the first constrained axis (RDA1). The partial redundancy analyses (pRDA) analyzed the relationship between outlier SNPs and climate variables when conditioned on geography

**FIGURE S4** Analyses of the SNP scaffold10517.2_56785 and its associated climate variable as well as adaptive phenotypic traits for the studied loblolly pine trees. (a) Geographical distribution of the SNP scaffold10517.2_56785 genotypes across counties of origin. (b) Variation in values of precipitation in May across counties of origin. (c,d) Geographical distribution of expression levels *of NCED* and *ANR* genes (normalized transcript levels based on stably expressed reference genes) across counties of origin
